# The Foundation of the Medico-Chirurgical Society and The History of Military Medicine in England

**Published:** 1915-12

**Authors:** G Parker

**Affiliations:** Major, R.A.M.C. (T.F.), 2nd Southern General Hospital; Physician to the Bristol General Hospital


### The Bristol Medico-Chirurgical Journal.

*"Scire est nescire, nisi id me

Scire alius sciret."*

524385

DECEMBER, 1915.

### THE FOUNDATION OF THE BRISTOL MEDICO-CHIRURGICAL SOCIETY, AND THE HISTORY OF MILITARY MEDICINE IN ENGLAND.

The Presidential Address, delivered on October 13th, 1915, at the opening of the Forty-second Session of the Bristol Medico-Chirurgical Society.

BY

G PARKER, M.A., M.D. Cantab.,

*Major, R.A.M.C. (T.F.), 2nd Southern General Hospital;
Physician to the Bristol General Hospital.*

FIRST of all I heartily thank the Society for the great honour it has conferred on me by electing me to this chair, an honour which I prize the more as it comes from old and valued friends whose kindness has shown itself in so many ways in the past. This year I shall have especial need of the support of every member, for never since its foundation has there been a time which called for greater care and anxiety among those responsible for the prosperity of this Society.

There are two subjects to which I should like to direct your attention to-night. One deals with the history of the Society as an embodiment of the medicine of our time. The other is the growth of the Royal Army Medical Corps, now claiming the minds and bodies of most of us, as one result of the development of military medicine in the past.

I would recall to your minds that it is forty years from the first Annual Meeting of the Bristol Medico-Chirurgical Society to the present one. The Society was designed primarily to help on our clinical and pathological work, and the circular letter which led to its foundation was signed by Dr. Shingleton Smith, Mr. Dobson and Dr. Spencer. There were then one hundred and forty medical men in Bristol and Clifton. There are now 275.

There were four hundred and forty-eight beds in the hospitals, and the population was about 196,000. It is now 363,000.

At this first Annual Meeting in February, 1875, it was announced that there were fifty members, an income of £26 5s. 0d., and an expenditure of £14 15s. 6d. The original President, Dr. Brittan, gave place then to Mr. Coë. Dr. Martin was to be Vice-President, and Dr. Shingleton Smith was the indefatigable Secretary, a position which he held till 1888.

After the election of officers a short business meeting took place, when Dr. Spencer discussed a case of cancer en cuirasse of the stomach, encephaloid disease as it was then called, with secondary growths. Dr. Martin was to have demonstrated prickle cells in rodent ulcer, and Mr. Dobson showed two calculi removed from children by the lateral operation. Eleven of the members of that year are alive, and twenty of those who appear in the first volume of *Transactions*, which covered the first four years of the Society's life.

Since the date of that meeting, now forty years ago, what changes have taken place in this Society, in our profession, and in the world. Since then we have established and kept going our *Journal* from 1883, which has reached its 128th number through the steady toil of our editorial staff. Since then too we have founded and collected a great library unparalleled in the West of England, about the future of which we have great hopes, though the present difficulties of it and the *Journal* are an anxious problem.

We can hardly realise in the steady current of our lives that the changes of medicine itself in that period have been gigantic. It may even be said that no other forty years in our history can show such advances. To take only two or three of them. There are, for instance, the development of aseptic surgery, and the elucidation of the diseases due to micro-organisms, *i.e.* the explanation at last of those strange disorders which run a limited career of their own and end of themselves.

The outburst of knowledge in these two subjects has altered the entire field of medicine and surgery more than the discoveries of any like period. No doubt the wonderful development of anatomy in the sixteenth century, the building up of physiology in the seventeenth by the aid of chemical, physical and mathematical science, the growth of pathological histology and the discovery of anæsthetics towards the middle of the nineteenth century equally aroused popular enthusiasm, and were great advances, but in the lifetime of our Society the greater part of our actual practice has been revolutionised by these two conquests.

It is interesting for us to note that in Dr. Brittan's inaugural address he took the line that a certain class of diseases are transmitted by particles. Dr. Fox rightly thought that was the most important position in it. Dr. Budd, of course, had gone on similar lines previously.

Davaine had discovered the anthrax bacillus in 1863. Pasteur had brought out some of his great results in 1864, and Lister commenced his work in 1865.

Our Society, then, began when the new teaching was being accepted, and its records reflect the great unfolding of the results.

There were illuminating addresses by Dr. Fripp on the contagium vivum, and by Dr. Fyffe on the poison of malaria in 1883, seven years before its nature was settled by the Campagna experiments and by the transmission of infected mosquitoes to London, where they reproduced the disease. Dr. Shingleton Smith started us with masterly expositions of the new theory of phthisis, and as soon as Koch demonstrated the bacillus in 1882, he presented the native product to our eyes, while Mr. Munro Smith showed us the presence of tuberculosis in the animal world. An outbreak of scarlet fever and one of infectious throats in Clifton gave opportunities for discussions which trained men in the new ideas. Mr. Greig Smith laid before us some of the new successes of surgery, and commenced a study of joint diseases which he did not live to complete. We find perhaps his earliest paper in the discussion on the treatment of pyrexia by antipyretic drugs or the cold bath. The advantages of the latter method as carried out by Brand and others included an actual saving of seven lives in every hundred in typhoid, a saving, by the way, which no one has shown to follow our more lazy method of sponging.

I must not stop to trace the great triumphs of antisepsis, of vaccines, and the huge technical work of pathological chemistry and bacteriology which have sprung up since 1875, and have led to some great successes and many failures.

A brilliant advance, too, in this forty years has been made in two other fields of disease, that of the nervous system and that of the heart. Both have been revolutionised since 1875.

Hughlings Jackson, Ferrier, Gowers and Ramon y Cajal have made a new map of the nervous system, and no one looking over the records of the Society can fail to be struck with the endless pathological work of Dr. J. Michell Clarke and others which has helped to build up the new structure. In more recent times the physiology and pathology of the heart has begun to be written anew through the labours of Mackenzie and others, in which some of our own members have also shared.

Laryngology, too, with the laryngoscope and great surgical operations on the throat and nose, and gynæcology, with its triumphs of ovariotomy and hysterectomy, are almost entirely changed. We look back with regret to the addresses in which Drs. Swayne and Aust Laurence dealt with the new advances.

Lastly, in public health we note a startling change both in organisation and in the science which earlier pioneers, such as Chadwick, had founded. The control of epidemic diseases, the cleansing of towns, the care of children and the practical abolition of typhus and typhoid are marvellous. I well remember at one of the first meetings I attended how Dr. Fox described the great typhus outbreak of 1865, and how it ceased to be infectious under treatment in huts outside the city.

The National Insurance Act, too, has aimed at supplying medical treatment to millions who were practically without it.

However, the enormous extension of these public services makes us tremble for the very existence of private practice. As Professor Morrison has remarked, how many patients are already provided for now by—

(1) The Navy, Army, Indian and Colonial Services.

(2) The Poor Law, with 238,000 persons out of doors, 108,000 indoors, and 67,000 in the infirmaries, and 125,000 lunatics.

(3) The Prisons, Reformatories, and Feeble-Minded Homes and Epileptic Colonies.

(4) The Tuberculosis Sanatoria and Dispensaries.

(5) The Infectious Diseases Hospitals with 25,000 beds, the Medical Officers of Health and their Inspectors.

(6) The Inspection and Treatment of School Children.

(7) The Inspection of Factories and Mines.

(8) The work of the Voluntary Hospitals.

(9) The fourteen and half millions treated by the Insurance Act.

Such a list makes us wonder whether in another half century there will be any private patients at all.

On the other hand, if we ask in which portion of our art least progress has been made in these forty years, we are struck with the few gains we have made in actual remedies for disease except surgical and preventive measures and a few vaccines and extracts. In the first volume of our *Transactions* is an elaborate paper by Dr. Spencer on the salicylates and salicin, then just coming into use. No drug probably of equal importance has been introduced in these forty years.

We had begun to lose faith in our drugs. It was already the fashion to belittle them. We in Clifton abandoned our hot wells with their strongly radio-active waters on which the prosperity of the place had arisen, and sent our patients for change of air to Buxton or Germany.

We have tried a countless host of new drugs since with scanty success, and I think we have lost much of the skill which our predecessors had in using old ones, such as opium.

Some of those who have lost all faith in them tell us that the very idea of pouring bottles of medicine into the body to effect a change in metabolism is absurd, but we daily see the lowly microbe producing these very changes by the secretions he pours in. It is a question of skill in the use of fit means for well-defined ends.

Still, the fact remains that we are dreadfully deficient in remedies for kidney disease, for gout, diabetes, rheumatoid arthritis, for the prevention of apoplexies, which destroy great hosts of people in the prime and fall of life, and for remedies against acute rheumatism, the universal malaria of the country, which ruins almost as many young lives as plasmodial malaria did in the past, and does in the tropics to-day. "Lord, have mercy on the poverty of our art," prayed an old physician.

A curious light is thrown on the inadequacy of our remedies by the enormous sale of patent medicines. It is easy to say that they are frauds and flourish on the superstition of the public, but whether they cure or not, they are bought because the buyers have pains which we often fail to relieve. Many of them are simple drugs thoroughly well compounded.

However, we urgently need an Act on the lines of the Select Committee forbidding fraud in their composition and the scandalous promises of cure for incurable diseases such as cancer and advanced phthisis, even if this entailed ruin on our Patriotic Press.

These matters are for the moment overshadowed by the pressing need of providing medical aid during the war for the civil population and for the Army. I shall therefore turn to the Royal Army Medical Corps and the history of military medical service in this country. The Corps has only existed in its present form from 1902, but organisations for the purpose appeared during our earlier wars.

Leaving out the thousand years from the coming of the Romans to William the Conqueror, our military annals in the last eight hundred and fifty years fall into four periods.

First, the period (1066 to 1314) of the consolidation of the

Anglo-Norman Empire includes numerous civil wars and certain crusades in the East.

Secondly, the period of the abortive attempt at expansion gives us the Hundred Years' War with France and the civil war which followed it (1348 to 1485).

Thirdly, the period (1515-1815) of the real expansion of England, the struggle for the New World and the building up of the Colonial Empire includes endless wars all over the world, and civil wars at home and in America.

The fourth period, from 1855 to 1915, shows a group of wars for the defence of the existing Empire, though expansion had not quite ceased.

To go back to the first group. As the armies were territorial, we may perhaps say that the onus of providing medical aid was laid on the local commanders. Little is known of the Norman medical service, except the names of one or two men like Dr. Nigel, who received lands at Clent and Droitwich for his services as an army surgeon with troops on the Welsh border; but there are other men attached to great nobles, such as Gregory, the doctor of the Earl of Warwick about 1120, Reginald of Bath, and Royal physicians and surgeons such as Grimbald, Ranulf of Besace under Richard I., Tyngewich and Weseham. The first instance of a Royal Medical Corps is seen in the group of seven physicians and surgeons whom Edward I. took north in his Scotch war of 1297. There were at first no Universities nearer than Italy where they could be taught, but in great hospitals such as St. Bartholomew's, in the commanderies of the Knights Hospitallers, in apprenticeships, and in many monasteries medical and surgical studies were carried on.

In our second period the new Universities, and especially the College of St. Como at Paris, had begun to train medical men, while in England certain strange legal corporations, the barber surgeons, gradually came to include almost the whole of the surgeons in the kingdom, and to give them in many instances a sound professional education by lectures, examinations and apprenticeships. It must be remembered that the surgeons in London, at any rate, became distinct from the barbers and others within the guild though united for legal purposes, just as in the great Florentine guild, of which Dante was a member, there were physicians, artists, mercers and thirty other callings. This official body afforded a reserve of surgeons. Thus in 1345 the city of York sent two barber surgeons to Bamborough to treat a political prisoner, David Bruce, and to extract an arrow from his wound, for which these gentlemen received a fee of about £50 each in our money.

For a time indeed the purely military surgeons had an organisation of their own, and as the French wars were carried out by well-paid troops and the ransom of prisoners was high, the health of both troops and prisoners made it worth while to give good fees to their doctors.

The most striking of these great army surgeons was John of Arderne, who, as Mr. D'Arcy Power says, was a scholar and a gentleman, a great traveller, who moved amongst the knights and heroes whose deeds in Flanders still live in the pages of Froissart.

One is charmed with his occasional chat about botany, the places where juniper grows in Kent and in Surrey, "where the people call it gorst because they do not know its proper name;" and his advice to the doctor to dress well and not like a minstrel, for, he says, a prudent man of clerkly dress and bearing, with clean hands and well-shapen nails may sit at any gentleman's table. He warns his young surgeon, too, to be courteous, gentlemanly and moral in word and deed. His works on surgery were popular text-books for ages. Perhaps one of the most striking things he wrote is a chapter on cancer of the rectum, the need of diagnosis by the skilled finger, the danger of mistaking it for dysentery, of operating upon it in error for something else, and the symptoms of the patient as he gets feebler and weaker, and sinks with a slight terminal fever. Whatever others may say, "I have never," he concludes, "seen or heard of any man who recovered from cancer, but I have known many who died of it."

An oft-quoted document shows how Henry V. agreed with a physician, Dr. Colnet, and a surgeon, Mr. Morstead, to take out a small medical corps to look after the army which fought at Agincourt five hundred years ago this month. Morstead's pay seems to have been about £1 16s. 0d. a day, with mess allowances and a share of booty or prize money. He was to have Mr. William Bredwardyne as lieutenant and twelve surgeons at 18s. a day in our money who were accordingly impressed.

I must not stop to talk of the medical guild which these military surgeons formed after the war—how they agreed not to steal each others patients, and to exclude for ever from practice chronic students who failed in two examinations; or about the wonderful Academy of Medicine and Surgery which they set up in London, and which came to an untimely end in 1423.

The third period commenced under the Tudors. Here we find the pay of military surgeons, about 1500 A.D., discussed by John Harvey, who says: —

"He that will be a surgeon in the wars must choose him as captain some noble liberal man that loves his men well, and must know what he will allow his surgeons a day. If he be a nobleman that is your captain, he will allow you as others do, 2s. [*i.e.* about £1] a day to the chief surgeon, unto the second 1s. 9d., to the third 1s. 4d., etc., and also a groat apiece of every soldier every month.

"His balderic must be of his master's colours about his neck with a spatula before, and behind with the king's arms in like manner. Besides [he can reckon on] the cases that he will have abroad among noblemen and other soldiers, if he be perfect in his science, well acquainted, gentle[manly], close, honest and merry. Also know what your master will allow you for your coffer [or military chest]. The captain will carry your chest, or else you must have a wagon with a horse or two amongst you, wherein you shall put your tents, your coffer, your bedstead and bed, your clothes, two or three shirts, two or three pairs of hose, your cassock or nightgown, your hood and hose of frieze, your high boots and ford boots, your various shoes and other things necessary for a surgeon."

In explanation of this we may note that the balderic was the officer's sash such as many of us remember, and from which the sword was formerly hung. The contents of the surgeon's chest were supposed to be provided from the 2d. or 4d. from each soldier, until Charles I. made a special grant.

During the Middle Ages, and until 1655, each company had its surgeon, but afterwards down to our own time he was a regimental officer. Thus in the army at St. Quentin, 1557, there was one surgeon to each one hundred men, the pay being 1s. a day in infantry up to 2s. in cavalry. The same pay seems to have been given to surgeons in the 70,000 men arrayed against the Armada and in the Irish campaigns.

The Elizabethan surgeons form a most brilliant group. Many of them had been trained under the rigorous educational system of the London Barber Surgeons Company, were well grounded in the new anatomy of Vesalius, and had served in Flanders and at Havre and elsewhere. Thus Will. Clowes, for instance, has left us a charming case book illustrating such matters as shot wounds, gunpowder burns, gangrene and wounds of the omentum. The old treatment of wounds by cleanliness and spirit lotions advocated by Mondeville had long given way to the use of endless ointments and lotions, but Clowes was a bold and careful surgeon. He would not accept the popular theory that all shot wounds were necessarily poisonous, though he got experiments made by the artillery commander at Portsmouth to find out whether an intentionally poisoned bullet was disinfected by the heat of the explosion and the passage through the air, and showed that there was no such effect.

In the case of a soldier who had an undiscovered bullet with a deep sinus in the groin for three years after a fight in Flanders, Clowes tried endless methods to detect and reach the bullet by flexible probes and dilators. As a last resource he bethought himself that a liquid would reach it, and injected through a long tube a solution of what seems to have been nitrate of silver and left it in. Next day pain and swelling appeared in the right buttock near the anus. He cut down on this, found and removed the bullet, and healed the sinus.

J. Gale, too, wrote on the old controversy upon the poisonous character of shot wounds, as well as an amusingly ferocious attack on the quacks of the time, the tinkers, pig-gelders, and out of work men whom Acts of Parliament failed to suppress. He says, too, that in one year he helped to recruit seventy-two surgeons in London for the fleet and army, but twenty years later there was a great shortage. Banister, the anatomist, was another prominent army surgeon who served both in Flanders and at Havre.

Peter Lowe, after many years in the continental wars, translated Hippocrates, and wrote a text-book of surgery. When he came home he persuaded the King of Scotland to found the existing Faculty of Physicians and Surgeons for Glasgow and district.

In the time of Charles I. the pay of the regimental surgeon rose to 2s. 6d. a day (money being worth three or four times its present value) and a free chest of stores to commence with, besides the twopence a month from each soldier.

The Parliament in the Civil War was much better off for money and better supplied with surgeons than the King, though both sides had often to call on local practitioners to help with the wounded.

Besides Physicians-General and Surgeons-General, with the oversight of an army, we find Staff-Surgeons both for the Staff and for hospitals. As to the latter, we are told that St. Bartholomew's, St. Thomas's, the Savoy, Ely House and Heriot's House in Edinburgh were used as Base Hospitals, while in 1652 Parliament sent two hundred and twenty invalids to Bath for treatment.

Before the Civil War old John Woodall, of St. Bartholomew's, after serving on the continent and travelling through Poland and Germany, became Surgeon-General to the new East India Company, and was sent to treat the wounded from the Isle of Ré. He has the honour of introducing lime juice for scurvy, and is the author of a text-book for naval surgeons called the *Surgeon's Mate*.

The incomparable Harvey can hardly be reckoned as an army surgeon, but the best surgical teacher of the time seems to me to be Alexander Read. His description of the circulation of the blood from the heart through the vessels and the lungs is derived apparently from Harvey, while his account of surgical sutures for the peritoneum, and of the use of ligatures, torsion and other means of stopping hemorrhage is remarkable. He describes, too, an experimental excision of the spleen.

The exemption of surgeons from serving on juries which had been granted to the London Barber Surgeons by Edward IV., and confirmed by the Great Statute of 1540, seems to have been given with a view to their being ready to serve in war. This is actually expressed in the charter of Queen Mary to the Edinburgh company. Accordingly one of the London Company's duties was to impress sufficient army surgeons in war time. Thus in 1626 they had to press surgeons for Rochelle and next year for the Isle of Ré, and sixteen more in 1628. In 1639 we find them pressing twenty-three surgeons for the Scotch war, and sending them by ship to Newcastle. In 1641 the justices impressed others for the Irish Rebellion, and in 1644 the Company again pressed men for the Parliament.

After the Restoration they had to find twenty surgeons and twenty assistants or mates for the war of 1672. We may add that their new charter of 1629, which gave the London Barber Surgeons the right to practise anywhere in England, had made the Company the examiner and licenser of all naval and ship surgeons. At one time they licensed five hundred men a year for the navy alone.

In the great Civil War two names of interest stand out on opposite sides, James Cooke, of Warwick, and Sir Richard Wiseman.

Cooke, the Puritan, had a good general practice, and served in the army of the Parliament. He is the man who went one day to see Shakespeare's daughter, good Mistress Hall, to examine the Latin medical manuscripts of her late husband, Dr. Hall, which he finally translated and published. She happened to say that one paper was not written by him, but confessed she could not read it. Possibly Cooke had some difficulty to make it out. It is upon this slender foundation that Baconians argue that Shakespeare's daughter, "witty beyond her sex," as her epitaph says, could not read or write.

Cooke wrote a most popular text-book of surgery, which went through endless editions, and a supplement on medicine, one of the rarest books in the world, where he discusses fevers, rickets, just made fashionable by Glisson, and small-pox, and ends with a discussion of what is needed in the surgeon's chest for the preservation of the wounded, and the care required in its use. "For," he adds, "the subject to be dealt withal is not a beast, but man, for whom, in some sense, the Son of God has shed His most precious blood, and if there be neglect it must be answered before the Lord at the dreadful day—for blood guiltiness in neglecting thy duty, or for both that and drunkenness [leading] to the ruin of men bearing perhaps a more fuller representation of God on them than you yourself."

Cooke is at his best in his accounts of cases he treated in the war. He tells us how two cavalry patrols mistook each other for the enemy near Kineton, and one of them got three bullets through his chest so that the air hissed in and out as he breathed. He was carried in a jolting cart to Warwick, some ten miles, but there he recovered without a check, Cooke wisely refraining from much interference.

He is rather proud of a case where he diagnosed a wound of the heart, which he verified *post-mortem*. It was a poor officer's servant who remonstrated with a soldier of the Scottish army when passing, and was stabbed in the side by a dirk for his pains. Cooke, standing at the gate of Warwick Castle, saw the man ride up blanched and breathless, and put him to bed, but felt the case was hopeless, for definite signs of a cardiac wound were present, and die he did in two days or less, but in a pious and edifying manner.

Sir Richard Wiseman, the Royalist, is an instance how a life of strenuous activity in the field may be combined with brilliant achievements in professional knowledge.

He had toiled in the rough school of the Dutch navy, and fought through the campaign of the Western Counties till he went into exile with Prince Charles. Again he served through the Scotch war of 1652 till the crowning disaster of Worcester. A prisoner in the Tower, and finally a naval surgeon in the Spanish fleet for three years, he came back to honour and wealth after the Restoration, to die at last at Bath, probably from hæmoptysis.

Who does not remember his war stories, such as that of the Irishman whose hand he amputated early one morning, and who beat him in a race two days after when the enemy surprised them both. He was a water drinker amongst wild-living cavaliers, and spent the leisure of his last years in writing a great classical work on surgery, "which is far beyond any previous work in building up general principles from the case under discussion."

After the Restoration the pay of army surgeons in the standing army was fixed at 4s. a day, and remained at that sum for a very long period. To each regiment a surgeon and his mate or assistant were allotted, and a grant was made to each company of £3 per annum for medicines and £3 for dressings. All candidates for the medical service had to get the approval of the Physician or Surgeon-General. The legal position of the army was at last defined by the Annual Mutiny Acts commencing in 1689. Previously it was difficult to enforce discipline in peace under existing statutes; and when five hundred of the Royal Scots deserted in a body and marched homewards the magistrates were helpless. It is under this Act, as renewed from year to year, that the King's Regulations, the soldiers' Bible as it has been named, has legal force.

In William III.'s Irish campaigns a fearful medical breakdown took place, and 6,300 men died from want and sickness, especially from dysentery, the disease of the country as it was called. To this breakdown we owe the first introduction of marching or field hospitals, clearing or fixed ones, and base hospitals such as Kilmainham in Dublin.

Under the care of Marlborough later on we find hospitals well maintained, and female nurses as well as male seem to have been employed.

Under the Hanoverian kings for the rest of this period the pay and the regimental system were both unchanged. The development of hospitals in three grades went on, while another most important step was taken in neutralising and protecting from capture medical officers and nurses with the sick and wounded by international conventions. Such agreements were made in 1743 and in the Seven Years' War. In the Napoleonic struggle the plan fell through, but at last by the Convention of Geneva, 1864, neutralisation became part of the public law of Europe.

I cannot now sketch the work of the great army physicians and surgeons of the later wars. Pringle, Grainger, Dimsdale and the officers of Wellington would require a lecture for themselves.

During the long peace after 1815 our medical system, defective as it was originally, fell into greater and greater disorder, in spite of the protests of the officers and of the Duke of Wellington himself. Even in peace the health of the soldier was neglected. He was enlisted for life, flogged mercilessly, and housed in such insanitary barracks that the death rate of the Foot Guards was twenty per thousand, while that of poor agricultural labourers of the same age was only eight, phthisis being the chief cause. The men slept on shelves, two together, round the barrack room. The beds were of straw down to a recent period, with a sheet of sacking washed once a month. The over-crowding would be inconceivable now. The soldiers' food and clothing were pilfered, not being protected by Government contracts. His body was unwashed and stinking. Only two meals a day were given him, but he had to drink a compulsory eight ounces of spirits.

The opinion of the medical officers went for nothing, for until the Royal Warrant of 1858, after the disclosures of the Crimea, they had no power even to make recommendations for the protection of the men's health. They might die, for instance, in bad barracks, such as the Up Park one in Jamaica, at the rate of one hundred and forty per thousand, while in the Maroon Town barracks close by the rate was only thirty-two, no medical opinion being asked or listened to. An old notice board from a door which has been preserved speaks volumes:

| |
|---|
| —TH REGIMENT. |
| COOKHOUSE, LAVATORY, & PRIVY. |

At last then this Warrant gave them not only the care of the sick and the administration of the hospitals, but also imposed on them the duty of recommending to the general and other commanding officers measures which may conduce to the health of the troops and to the prevention of disease.

This was a great step, but the doctors were isolated and feeble individuals in each regiment. Each of them had a tiny hospital and three soldiers as orderlies. Even here the medical officer had no power of command, and the sergeant maintained discipline.

The system was hopeless in war. There were forty-seven such dolls' hospitals in an army corps, but no ambulance, or organisation for carrying off the wounded. One battalion might have hundreds of wounded with only one surgeon and no trained dressers, nurses or transport; others might have their surgeons standing idle.

The system failed, as Evatt says, on the Alma hillside in September, and came to utter grief in the corridors of the great Scutari hospital in the winter. Our whole organisation is based on the bitter lessons learned then. In a word we needed the means of removing the sick and wounded in comfort instead of carrying them on with the fighting line, which is impossible in a rapidly-moving modern army.

When general hospitals were formed eight or nine different departments governed them, but no single head compelled co-operation, as Miss Nightingale remarked.

There was no uniform entrance examination for surgeons, no practical tests of efficiency or of knowledge of preventive medicines for the very men who had to safeguard our troops in every part of the world.

Two popular movements gave a great impetus to the improvement of the service. One was the rapid development of the art of nursing which followed Miss Nightingale's splendid work in the Crimea. This led in time to the introduction of nursing sisters and the thorough training of nursing orderlies. Again, the rise of Voluntary Aid Societies for helping the wounded after the Geneva Convention, and still more in the Franco-German War of 1870, produced a huge reserve of trained bearers and transport.

After all the efforts of Sidney Herbert and other reformers it was not till 1873 that the old regimental system was broken up, and the medical officers were formed into one body, the Army Medical Department, and the nursing orderlies were made into the Army Hospital Corps. Four years later the actual command of this corps and of the general hospitals was given to medical men.

The Ambulance first appeared as stretcher bearers in 1873, and bearer companies in the Transvaal War of 1879–80. Field hospitals were formed in Egypt in 1882. Other changes had to wait, and in 1886 the Army Medical Department was cut down for economy. Further improvement of the medical service seemed as far off as ever. The officers were isolated from the activity of civil professional life on the one hand and from the *esprit de corps* of the combatant ranks on the other. In spite of the brilliant work of such leaders as Ronald Ross, Leishman and Bruce, the service attracted fewer and fewer men.

In 1897 fresh reforms were promised, and Lord Lansdowne obtained from Queen Victoria a Warrant constituting the Royal Army Medical Corps, but it had few attractions, and candidates were fewer than the vacancies.

At the outbreak of the Boer War there were only five hundred and forty medical officers to supply the needs of the depots and of an army of 250,000 men, one-third or a quarter of the proper number. The terrible breakdown which resulted in Africa, and the losses due to the soldiers' ignorance of sanitation, as well as to the deficiency of medical officers, led again to a great public inquiry, and finally Mr. Brodrick's Re-organisation Committee and Civilian Advisory Board carried out complete and searching reforms.

The Royal Warrant of 1902 and the following Orders end our story happily, for they not only granted improved pay, but they gave substantive rank, they authorised an increase in the numbers, which made it possible to grant leave for study and research. Under them the grand new Royal Army Medical College for study was built at a cost of £100,000 in London instead of at Netley, so that the best opportunities for scientific work might be available. Under them higher posts are given as rewards for special proficiency or by practical examination, and not by seniority. Under them the study of sanitation was placed in the highest rank, and elementary teaching in it is given not only to the privates of the corps, but also to combatant officers of the line.

The deficiency of candidates ceased; even in that year the medical officers rose to 965, and plans were drawn up for great Territorial reserves, through which some of our present armies have been staffed, while the National Red Cross Society and the Association formed by the Order of St. John were entrusted with the preparation of a host of additional bearers, nurses and hospitals.

In conclusion, the success of the present organisation, preserving the lives and health of our soldiers in the last twelve months, is acknowledged on all sides, and we may look forward to even greater triumphs in the future.

NOTE.—I gratefully acknowledge the help I have received from the papers of Howell, Evatt and other writers in the Journal of the R.A.M.C.

### CARDIAC DISEASES AND DISORDERS IN WARFARE.

